# Multiplex detection of five common respiratory pathogens from bronchoalveolar lavages using high resolution melting curve analysis

**DOI:** 10.1186/s12866-022-02558-2

**Published:** 2022-05-19

**Authors:** Jaber Ghorbani, Farhad Bonakdar Hashemi, Fereshteh Jabalameli, Mohammad Emaneini, Reza Beigverdi

**Affiliations:** 1 Department of Microbiology, School of Medicine, Tehran University of Medical Sciences, Building No. 6, 100 Poursina St., Keshavarz Blvd., Tehran, Iran; 2 Medical Mycology and Bacteriology Research Center, Kerman University of Medical Sciences, Kerman, Iran

**Keywords:** Lower respiratory tract infection, Bronchoalveolar lavage, Rapid detection, HRM assay, Melting curve

## Abstract

**Background:**

The study describes the application of the multiplex high-resolution melting curve (MHRM) assay for the simultaneous detection of five common bacterial pathogens (*Pseudomonas aeruginosa*, *Staphylococcus aureus*, *Klebsiella pneumoniae*, *Acinetobacter baumannii* and *Escherichia coli*) directly from bronchoalveolar lavage samples.

**Results:**

Our MHRM assay successfully identified all five respiratory pathogens in less than 5 h, with five separate melting curves with specific melt peak temperatures (Tm). The different Tm were characterized by peaks of 78.1 ± 0.4 °C for *S. aureus*, 83.3 ± 0.1 °C for *A. baumannii*, 86.7 ± 0.2 °C for *E. coli*, 90.5 ± 0.1 °C for *K. pneumoniae*, 94.5 ± 0.2 °C for *P. aeruginosa*. The overall sensitivity and specificity of MHRM were 100% and 88.8–100%, respectively.

**Conclusions:**

Our MHRM assay offers a simple and fast alternative to culture approach for simultaneous detection of five major bacterial lower respiratory tract infection pathogens. Utilization of this assay can help clinicians initiate prompt and appropriate antimicrobial treatment, towards reducing the morbidity and mortality of severe respiratory infections.

**Supplementary Information:**

The online version contains supplementary material available at 10.1186/s12866-022-02558-2.

## Background

Lower respiratory tract infections (LRTIs) are the fourth leading cause of death around the world, responsible for 2.38 million deaths annually [1, 2]. Nosocomial pneumonia or hospital acquired pneumonia (HAP) is a major health problem in many hospitals of both developed and developing countries [3]. HAP is most often caused by both Gram-positive and Gram-negative bacteria. The most common bacteria implicated in the development of HAP are *Pseudomonas aeruginosa, Staphylococcus aureus, Klebsiella pneumoniae, Acinetobacter baumannii* and *Escherichia coli* [4]. These pathogens are often resistant to various antibiotics, making LTRIs caused by them difficult to treat [5, 6]. It is clear that a fast, simple and accurate diagnosis for the detection of LRTI pathogens is vital to the selection of antibiotic therapy and management of patient treatment [7]. The current standard for LRTIs is bronchoalveolar lavage (BAL) culture, but this is time-consuming, labor-intensive and has low sensitivity, particularly when the patient has been given antibiotic therapy prior to sampling [7–9]. For these reasons, molecular methods have been developed to improve the diagnosis of bacterial respiratory infections. One of these methods is high-resolution melting (HRM) analysis, which relies on a real‐time PCR method in the presence of the double stranded DNA (dsDNA) intercalating fluorescent dye and monitors changes in melting of dsDNA with increasing temperature [10–12]. HRM has been used for genotyping, detection of bacterial resistance genes, as well as for detection and differentiation of various pathogenic organisms such as bacteria, fungi, and parasites using primers targeted at conserved regions within ribosomal gene [13–19]. Although targeting 16S rRNA has shown promising results, this approach requires further analysis for definitive identification and is complicated in multiplexed designs [20]. A recent study developed a multiplex HRM assay using species-specific primer sets for speciation of most common Gram-negative pathogens [21]. However, this method has not been applied directly on BAL samples to simultaneously distinguish several pathogens. In this study we have developed a multiplex HRM (MHRM) assay using species-specific primers for the simultaneous detection of five common bacterial pathogens (*P*. *aeruginosa, S*. *aureus, K. pneumoniae, A. baumannii* and *E. coli*) directly from BAL samples.

## Results

### MHRM speciation assay

**Fig. 1:**
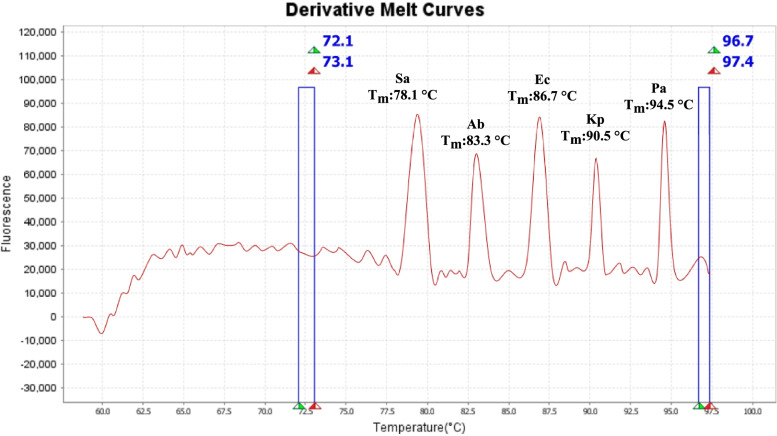
Discrimination of five Mixed-Species DNA samples from pure culture targeted by High-Resolution Melting (HRM) analysis. Each species indicated a unique melting temperature in one single reaction. Ab (*A. baumannii*), Kp (*K. pneumoniae*), Pa (*P. aeruginosa*), Sa (*S. aureus*), Ec (*E. coli*)

**Fig. 2:**
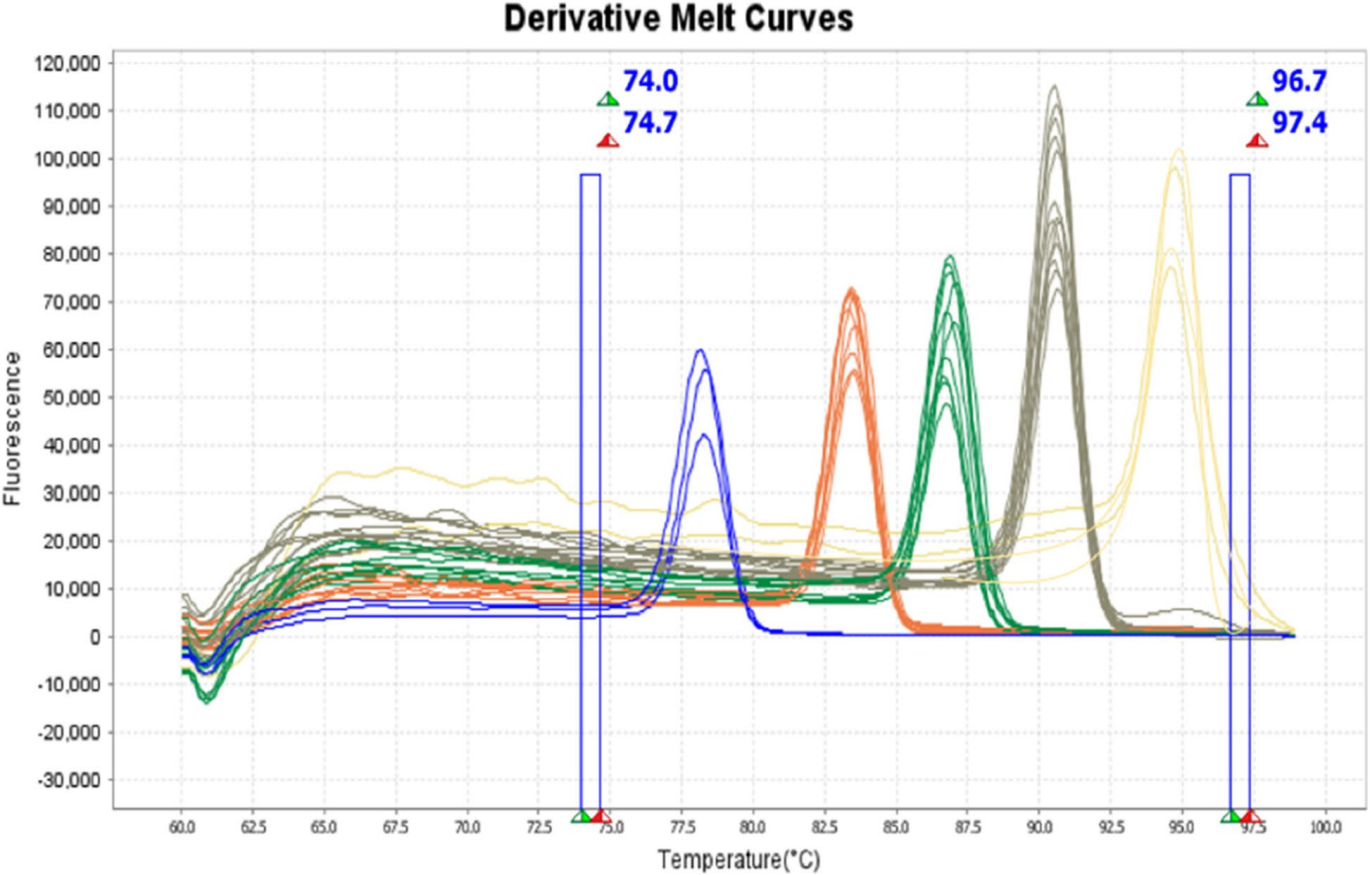
MHRM profiles of clinical samples (BAL) (*n* = 34) from patients with suspected pneumonia and cultured standard isolates (n = 5). The same colors illustrate the same pathogens. Blue curve (*S. aureus*) (n = 3); orange curve (*A. baumannii*) (*n* = 9); Green curve (*E. coli*) (*n* = 7); gray curve (*K. pneumoniae*) (*n* = 11); yellow curve (*P. aeruginosa*) (*n* = 5)

**Fig. 3:**
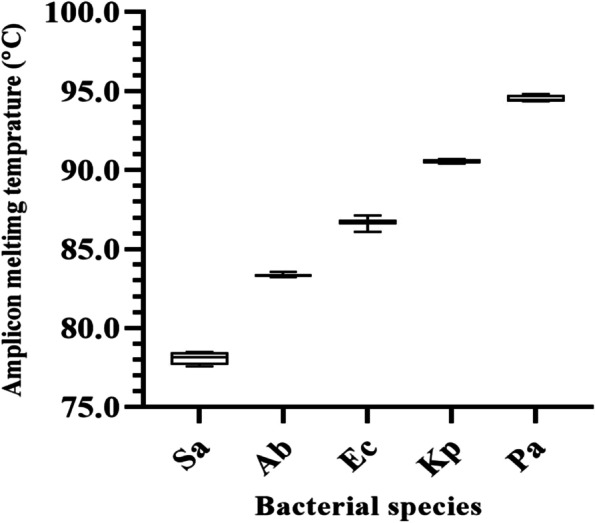
Box plot showing the variation ranges of melting temperature of each pathogen in clinical samples. The ANOVA test showed significant difference of average Tm values between pathogens (*P* = *0.001*). Sa (*S. aureus*), Ab (*A. baumannii*), Ec (*E. coli*), Kp (*K. pneumoniae*), Pa (*P. aeruginosa*)

Our results indicate that the MHRM assay designed in this study was able to distinguish standard cultures of all five bacterial species. Figure 1 shows that MHRM assay is capable of detecting the combination of all five pathogens, which were artificially created in the lab, in one single reaction. In addition, MHRM identified clinical BAL samples containing a mixture of 2–3 pathogens (Supp. Figure 1). Figure 2 illustrates the unique pattern of the derivative and aligned melting curve which demonstrates a unique Tm for each bacterial species that helps differentiate all five test bacterial species from clinical samples. The melting curves were characterized by peaks of 78.1 ± 0.4 °C for *S. aureus* (n = 9), 83.3 ± 0.1 °C for *A. baumannii* (n = 25), 86.7 ± 0.2 °C for *E. coli* (n = 6), 90.5 ± 0.1 °C for *K. pneumoniae* (n = 25), 94.5 ± 0.2 °C for *P. aeruginosa* (n = 11). The overall Tm ranges with each of the pathogens are illustrated in Fig. 3. The assay was repeated several times on different days by the same researcher and reproduced by other researchers in the lab.

### MHRM limit of detection and specificity

The limit of detection (LOD) of MHRM assay for *A. baumannii, P. aeruginosa, K. pneumoniae and E. coli* was between 0.8–1 × 10^3^ CFU/ml, and for *S. aureus,* it was 1.3 × 10^3^ CFU/ml (Supp. Figure 2). Data revealed that MHRM assay specifically detected all 5 test pathogens, without non-specific amplifications, when DNA from human cells or other species of bacteria were used as template.

### Comparison of MHRM assay to culture identification of pathogens

Of 96 BAL specimens analyzed, 35 specimens (36.4%) were concordantly negative by culture as well as MHRM. The detection rate of MHRM was higher than that of culture, since 54 (56%) of samples were culture positive, whereas 61 samples (63.5%) were positive by MHRM assay (*P* < 0.001). Table 1 compares the list of identified pathogens recovered by both culture and MHRM, and demonstrates that all culture-positive samples were also MHRM positive, with identical species identification (Table 1). *A. baumannii* (23%, 22/96) was the most common pathogen, as determined by both culture and MHRM assay. In five samples out of 96, two melting peaks were observed in the MHRM derivative plots, suggesting the presence of two pathogens (three samples: *E*. *coli* and *A. baumannii* and two samples: *P. aeruginosa* and *K. pneumoniae*); however, culture identified one pathogen (Table 2). Two samples out of 96, showed three melting peaks in the MHRM derivative plots, suggesting the presence of three pathogens (one sample: *A. baumannii, P. aeruginosa* and *K. pneumoniae* and one sample: *A. baumannii, K. pneumoniae* and *S. aureus)* (double and triple-bacterial melt curves is shown in Supp. Figure 1). However, the culture method identified one and two pathogens for each sample, respectively (Table 2). Compared to culture, the specificity of the MHRM ranged from 88.8% to 100%, and sensitivity 100% for all test pathogens (Table 2).

**Table 1:** Comparative analysis of HRM and culture identification

Infection level of BAL specimens No. (%)	HRM	Culture	Frequency No. (%)
Single bacterial infection *N* = 48 (50%)	*A. baumannii*	*A. baumannii*	14 (14.6)
	*K. pneumoniae*	*K. pneumoniae*	12 (12.5)
	*P. aeruginosa*	*P. aeruginosa*	7 (7.3)
	*S. aureus*	*S. aureus*	6 (6.3)
	*E. coli*	*E. coli*	2(2.1)
	*K. pneumoniae*	*-*	4 (4.2)
	*A. baumannii*	*-*	2 (2.1)
	*P. aeruginosa*	*-*	1 (1)
Double bacterial infection *N* = 11 (11.5%)	*A. baumannii* *E. coli*	*A. baumannii*	2(2.1)
	*A. baumannii* *S. aureus*	*A. baumannii* *S. aureus*	1(1)
	*A. baumannii* *K. pneumoniae*	*A. baumannii* *K. pneumoniae*	3(3.1)
	*A. baumannii* *E. coli*	*E. coli*	1(1)
	*E. coli* *K. pneumoniae*	*E. coli* *K. pneumoniae*	1(1)
	*P. aeruginosa* *K. pneumoniae*	*P. aeruginosa*	2(2.1)
	*K. pneumonia* *S. aureus*	*K. pneumonia* *S. aureus*	1(1)
Triple bacterial infection *N* = 2 (2.1%)	*A. baumannii* *K. pneumoniae* *P. aeruginosa*	*A. baumannii*	1(1)
	*A. baumannii* *K. pneumoniae* *S. aureus*	*A. baumannii* *S. aureus*	1 (1)
Negative specimens *N* = 35 (36.4%)	None	None	35(36.4)

**Table 2:** Comparison of sensitivity and specificity of MHRM assay versus culture identification method, and the degree of agreement between the methods for each test pathogen

Target	True Positive	True Negative	False Positive	False Negative	Sensitivity (%)	Specificity (%)	Agreement (%)	Cohen’s Kappa
*A. baumannii*	22	71	3	0	100	97.3	96.9	0.92
*K. pneumoniae*	17	71	8	0	100	88.8	91.6	0.76
*P. aeruginosa*	9	85	2	0	100	97.7	97.9	0.89
*S. aureus*	9	87	0	0	100	100	100	1.00
*E. coli*	4	90	2	0	100	97.8	92.7	0.79

## Discussion

The rapid and accurate identification of LRTIs is critical for appropriate antimicrobial therapy, which is strongly associated with positive clinical outcomes [7, 8]. Different diagnostic molecular methods have been introduced to identify lower respiratory tract pathogens. These molecular techniques are typically used to identify bacteria from primary cultures which is much faster than second culture and biochemical tests. 16S rRNA gene sequencing is a precise and commonly used method for detection and identification of bacteria; however, is pricey and time consuming [22]. The multiplexed commercial microbiological assays including Biofire designed for use with respiratory panel (RP) or VERIgene system (NanoGrid Technology) can identify bacteria from primary culture in about an hour; however, need appropriate piece of equipment that are hardly available and expensive [7, 23]. VITEK 2 microbial identification system which is available in some laboratories can provide results within about 5 h; however, requires single colony from primary pure culture of microorganism and is also costly [24]. In recent years, probe-based assays have been developed for detection of different bacteria. However, probes are expensive and complex to synthesize. Moreover, previous studies have shown that probe-based real-time PCRs are limited by failure to distinguish bacteria in multiplexed experiments [21, 25]. In this study, a MHRM assay has been successfully developed for rapid and accurate identification of five common respiratory bacterial pathogens directly from BAL specimens. The use of species-specific primer sets provides unambiguous results, which is easy to interpret and does not require highly trained microbiologists to identify bacterial species. The test also provides the results within less than 5 h, including sample preparation, DNA extraction and HRM analysis, which is considerably shorter than the time required for preparing pure culture in culture-based methods. The type of dye plays a significant role in fluorescence melting curve analysis. Compared to first generation dyes like SYBR Green, EVA Green has a greater sensitivity than SYBR Green in multiplex designs, which can give more reproducible results [26]. In our study, third generation saturating dye, EVA Green was applied as dye. Our results indicate that MHRM assay is highly specific as all targets were identified accurately, with no fluorescence signal detected in samples containing non-target DNA. The melting temperature for DNA from each target pathogen was sufficiently different (ideally > 1 °C), to enable simultaneous discrimination among all pathogens in BAL sample. The melting curves produced by the clinical specimens showed consistency in their Tm without much shift from those observed using standard isolates. Quantification of the bacteria in the LRTIs is the key to differentiate between colonization and true infection [27]. In this study, the LOD of the MHRM assay ranged between 8 × 10^2^ to 1.3 × 10^3^ CFU/ml for different target pathogens, which is lower than the number of bacteria suggested for identification of bacterial infection via culture (10^4^ CFU/ml). Therefore, samples which are reported as culture negative and MHRM positive cases can be explained as contamination or colonization. Some of the discrepant results (MHRM-positive but culture-negative) observed in this study lies in the fact that molecular assays can amplify the DNA from dead organisms, resulting in clinically false-positive results [28]. One of the main drawbacks of the MHRM assay is its inability to accurately quantify the number of pathogens in samples, hence clinicians cannot judge that the sample is from an infection or colonization. Therefore, a positive result by MHRM assay should be carefully analyzed, considering clinical symptoms, chest radiograph findings and other laboratory tests, such as CRP and WBC results [29]. Considering the fact that coinfections with a mix of 2–3 bacteria can occur in lower respiratory tract infections [29, 30], and current rapid methods have poor sensitivity for identifying all species in mixed samples, therefore, the ability of the assay for detection of mixed infections is considerably important. The results of multi-species spiked samples indicated that the MHRM assay has the potential to detect coinfection with more than one pathogen. However, the results from clinical samples revealed that the sensitivity of the MHRM assay in detection of double or triple-bacterial clinical samples is lower than the sensitivity in detection of mono bacterial infections (Table 1). In this study, the MHRM assay showed more than 88% specificity and 100% sensitivity for each pathogen. Another MHRM method developed by Edwards et al. reported an overall sensitivity of 97.1% and a specificity of 100% for detection of six common Gram-negative pathogens [21]. This difference may be related to the fact that we extracted DNA directly from the BAL samples, but Edwards et al. extracted DNA from the pure cultures or single colonies. One major limitation of MHRM in this study that could be addressed in future research is lack of internal amplification control (IAC). An IAC can be used as an effective tool to provide assurance that clinical specimens are successfully amplified and detected. Though our MHRM showed no false negative results as compared to the culture, lack of IAC suggests that the test has to be repeated, for instance using new reagents or an alternative method of DNA extraction and purification.

## Conclusion

We have developed a MHRM assay that may be used as a functional tool for the diagnosis of LRTIs through detection of the potential pathogen directly from the clinical BAL samples. By offering an accurate simultaneous identification of the causative agent(s) of LRTI within a timeframe much shorter than culture method MHRM can help clinicians to initiate timely and appropriate antimicrobial therapy, and hence reduce morbidity and mortality associated with LTRIs.

## Materials

### Study design and identification of isolates

A total number of 96 BAL specimens were collected from hospitalized patients with suspected pneumonia from four hospitals (Tehran, Iran) during May 2018-January 2019. All specimens were cultured on the 5% sheep blood, chocolate, MacConkey agar plates and incubated at 37 °C overnight. Bacterial colony counts and species identification were carried out according to validated standard operation procedures [23, 27, 29, 31]. BAL cultures were considered positive if 10^4^ bacteria or more per milliliter of BAL were found [23, 29].

### DNA extraction

DNA was directly extracted from BAL specimens and standard isolates using the FAVOGEN DNA Extraction Kit (Biotech Corp, Taiwan), following the protocol for extraction. Concentration and purity of extracted DNA were determined by a Nanodrop® 2000c instrument (Thermo Fisher Scientific, USA). DNA was kept at -20 °C for future experiments.

### Primer designing for the study

The whole genome sequences of *P*. *aeruginosa* (GenBank accession number CP050332), *K*. *pneumoniae* (GenBank accession number CP077773), *S*. *aureus* (GenBank accession number CP053639), *A*. *baumannii* (GenBank accession number CP000521), and *E*. *coli* (GenBank accession number CP034658) were downloaded from NCBI database. Comparative analysis of the chromosomes of these five species was performed in order to identify conserved regions (species-specific sequences). Five primer sets were designed using an online primer3 software (http://primer3.ut.ee/) and the specificity of primers was assessed in silico using primer-BLAST program available at https://www.ncbi.nlm.nih.gov/tools/primer-blast/. The theoretical Tm of amplicons was calculated with Oligonucleotide Properties Calculator (OligoCalc) based on the amplicon sequence (http://biotools.nubic.northwestern.edu/OligoCalc.html). The five primer sets generated 153 to 272 bp products and the sequences of the forward and reverse primer, their targets, and predicted amplicon Tm are presented in Table 3. The efficiency of primers was assessed by using conventional PCR. A set of conventional gradient PCR was performed to ensure that the five pairs of primers were able to amplify the target region in the five species without producing unspecific PCR products or primer dimers that interfere with the interpretation of results in PCR-HRM analysis later on. The reaction mixture contained 12.5 μL PCR Master Mix 2X (Ampliqon, Denmark), 0.5 μL of each primer (10 pmol, Metabion, Martinsried, Germany), 1 μL of DNA (10–20 ng/μL) and 10.5 μL of DNase-free water in a total reaction volume of 25 μL per sample. The PCR thermocycling conditions consisted of an initial denaturing step at 95 °C × 50 s, followed by 30 cycles of denaturation at 95 °C × 15 s, annealing from 56 °C to 65 °C × 20 s, extension at 72 °C × 20 s and a final extension at 72 °C × 10 min. The reactions were performed in a T100™ thermal cycler (Bio-Rad). The amplified DNA fragments were electrophoresed in a 1.5% agarose gels with 0.5X TBE (Tris/Borate/EDTA) buffer. The DNA bands were visualized by KBC power load dye staining and photographed under UV illumination.

**Table 3:** Comparison of sequence, amplicon size, GC content and melting tempreture of specific PCR primers used for each pathogen in the MHRM analysis. List of primers used in this study and its properties

Pathogen	Gene Bank accession no	primer sequence/ Tm (°C)	Nucleotide positions	Amplicon size (bp)	GC content of amplicon (%)	Predicted Tm^a^ (°C)	Observed Tm^b^ (°C)
*A. baumannii*	CP000521	F: GTGGCACATTAGGTCCCGA (56.4) R: CAAGGTAGTCTGCTTGAGTCG (58.4)	3,143,607- 3,143,795	189	44	82.09	83.33
*K. pneumoniae*	CP077773	F: GGCGAGGTTTACGTCTCAAC (55.9) R: GTACTTCTTGTTGGCCTCGC (56.2)	5,129,431- 5,129,702	272	61	89.34	90.59
*P. aeruginosa*	CP050332	F: ATCTTCTGGCTGTCTTCGGC (55.3) R: AATGTCCACCACGGTCTTCC (56.3)	2,396,197- 2,396,388	192	70	92.77	94.57
*S. aureus*	CP053639	F: GCTAAACCACTTTTGTTAGCACC (58.7) R: TGATAAAGAAAATGGCATGCACA (57.6)	1,867,646- 1,867,798	153	31	76.44	78.1
*E. coli*	CP034658	F: CATACCTGTTCACCGACGAC (55.4) R: CTGGCAGGAGAAACTGCATC (56.1)	1,662,333- 1,662,506	174	53	84.95	86.74

^a^Amplicon melt point calculated by OligoCalc program

^b^Amplicon melt point calculated by ABI StepOnePlus Real-Time PCR instrument

### Multiplex HRM-real-time PCR assay

Multiplex real-time PCR with HRM (MHRM) analysis was performed sequentially on a ABI StepOnePlus Real-Time PCR (Applied Biosystems) in a reaction mixture containing 4 µl of 5 × HOT FIREPol® EvaGreen® HRM Mix no ROX (Soils Biodyne, Estonia), 0.5 μL of pathogen-specific primer (10 pmol, Metabion, Martinsried, Germany), 1 μL of DNA (10–20 ng/μl) and 14 μL of DNase-free water in a total reaction volume of 20 μL per sample. Positive controls (containing genomic DNA from each species) and negative controls (DNase-free water) were included in each run. The reaction conditions involved enzyme activation at 95 °C × 15 min, followed by 40 cycles of denaturation at 95 °C × 15 s, 63 °C × 20 s for annealing, and 72 °C × 20 s for extension. Following this, HRM was carried out by heating the mixture from 60 °C to 99 °C using a ramping degree of 0.3 °C/sec. The melt curve analysis was carried out using HRM Software version 3.0.1(Applied Biosystems).

### Limit of detection

The LODs of MHRM assay were initially determined using 11 concentrations of each isolate, preparing in 0.5 McFarland (Supp. Figure 2). A 10 µl sample of each dilution was plated and the colonies were counted to determine the CFU/ml in each dilution. Genomic DNA was extracted from all dilutions by DNA Extraction Kit (Biotech Corp, Taiwan), according to the manufacturer’s instructions. The specificity of the MHRM assay was tested using various DNA from other organisms such as human genomic DNA, *Staphylococcus epidermidis*, *Salmonella* Typhimurium*, Streptococcus pneumoniae, Haemophilus influenzae, Proteus mirabilis* and *Streptococcus pyogenes*. We calculated the sensitivity and specificity of the real-time PCR with MHRM analysis for the detection of five common bacterial pathogens compared with culture, which was used as the reference standard.

### Detection of multi-species spiked samples

In order to assess the ability of MHRM to detect infections with more than one pathogen, extracted DNA from each of the *P. aeruginosa, S. aureus, K. pneumoniae, A. baumannii* and *E. coli* were spiked with DNA of other two, three or four above mentioned pathogens. Consequently, 1 µl of bacterial DNA combinations were tested by MHRM assay.

### Statistical analysis

The sensitivity and specificity of the MHRM assay was determined by comparison to culture, as the reference standard method. Kappa correlation was utilized to assess the degree of agreement between the two methods. All statistical analyses were performed using the Statistical Package for the Social Sciences (SPSS®) software (version 21, IBM Corp.).

## Supplementary Information

**Additional file 1: Supp. Fig 1.** The representative meltingcurves of two patient samples showing major peaks with differential T_m_values. (A) MHRM graph of a specimen with double bacterial infection; showing twosignificant peaks, which signify the presence of Sa (*S. aureus*), and Pa (*P. aeruginosa*), and (B) MHRM graph ofa specimen with triple bacterial infection; including Sa (*S. aureus*), Ab (*A. baumannii*),and Kp (*K. pneumoniae*) showing three significant peaks. **Supp.Fig 2**. The limit of detection of MHRM. (A) *S.aureus*; (B) *E. coli; *(C)* A. baumannii; *(D)* K. pneumoniae; *(E)*P. aeruginosa.* a (1.5×10^7^); b (1.5×10^6^); c (1.5×10^5^);d (1.5×10^4^); e (1.5×10^3^); f (1.25×10^3^); g (1.0×10^3^);h (0.75×10^3^); i (0.5×10^3^); j (1.5×10^2^); k(1.5×10^1^); NTC (Non-template negativecontrol).

## Data Availability

The datasets used and/or analyzed during the current study are available from the corresponding author on reasonable request. Most of the data is included in this article [and its Additional file (Supp. Figures 1 and 2)].

## Abbreviations

LRTIs: Lower Respiratory Tract Infections
HAP: Hospital Acquired Pneumonia
BAL: Bronchoalveolar Lavage
HRM: High-Resolution Melting
Tm: Melting Temperature
dsDNA: Double Stranded DNA
MHRM: Multiplex HRM
LOD: Limit Of Detection
