# Supplementary material for: Multiplex detection of five common respiratory pathogens from bronchoalveolar lavages using high resolution melting curve analysis
Source: BMC Microbiol. 2022 May 19;22:141. doi: 10.1186/s12866-022-02558-2 (PMC9118692; doi:10.1186/s12866-022-02558-2)
Supplement: Supplementary file 1 — Additional file 1: Supp. Fig 1. The representative meltingcurves of two patient samples showing major peaks with differential Tmvalues. (A) MHRM graph of a specimen with double bacterial infection; showing twosignificant peaks, which signify the presence of Sa (S. aureus), and Pa (P. aeruginosa), and (B) MHRM graph ofa specimen with triple bacterial infection; including Sa (S. aureus), Ab (A. baumannii),and Kp (K. pneumoniae) showing three significant peaks. Supp.Fig 2. The limit of detection of MHRM. (A) S.aureus; (B) E. coli; (C) A. baumannii; (D) K. pneumoniae; (E)P. aeruginosa. a (1.5×10^7^); b (1.5×10^6^); c (1.5×10^5^);d (1.5×10^4^); e (1.5×10^3^); f (1.25×10^3^); g (1.0×10^3^);h (0.75×10^3^); i (0.5×10^3^); j (1.5×10^2^); k(1.5×101); NTC (Non-template negativecontrol). [file 12866_2022_2558_MOESM1_ESM.docx]

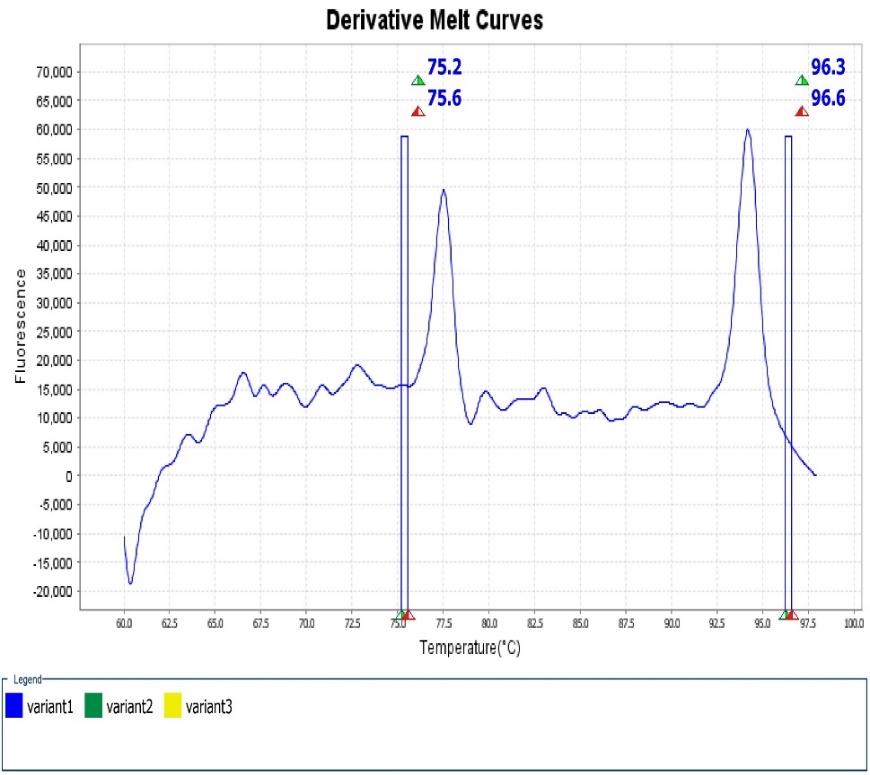

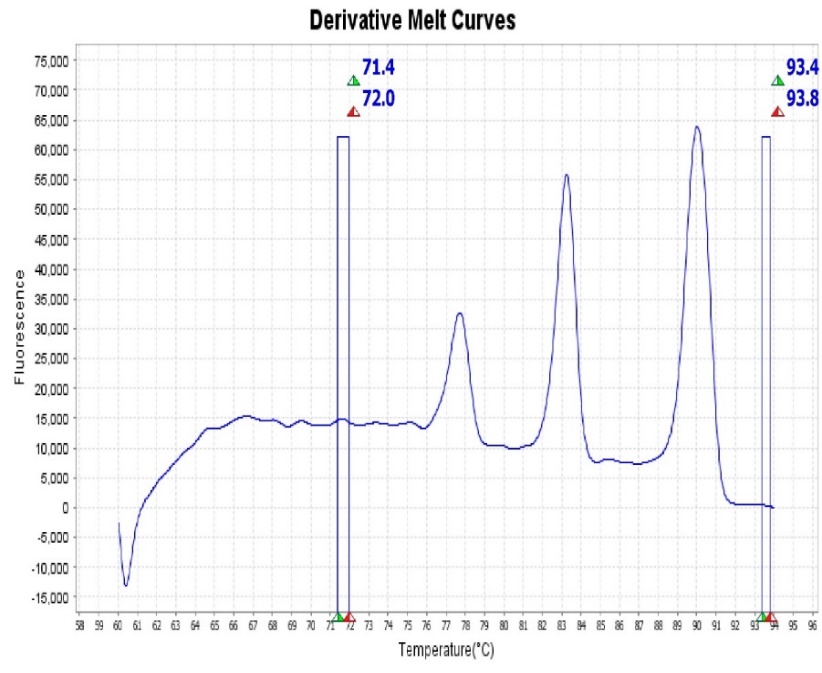


Kp

Tm:90.5°C

Ab

Tm:83.3°C

Sa

Tm:78.1°C

Sa

Tm:78.1°C

Pa

Tm:94.5°C

A

B

**Supp. Fig 1.** The representative melting curves of two patient samples showing major peaks with differential T_m_ values. (A) MHRM graph of a specimen with double bacterial infection; showing two significant peaks, which signify the presence of Sa (*S. aureus*), and Pa (*P. aeruginosa*), and (B) MHRM graph of a specimen with triple bacterial infection; including Sa (*S. aureus*), Ab (*A. baumannii*), and Kp (*K. pneumoniae*) showing three significant peaks.

**Supp. Fig 2**. The limit of detection of MHRM. (A) *S. aureus*; (B) *E. coli;* (C) *A. baumannii;* (D) *K. pneumoniae;* (E) *P. aeruginosa.* a (1.5×10^7^); b (1.5×10^6^); c (1.5×10^5^); d (1.5×10^4^); e (1.5×10^3^); f (1.25×10^3^); g (1.0×10^3^); h (0.75×10^3^); i (0.5×10^3^); j (1.5×10^2^); k (1.5×10^1^); NTC (Non-template negative control).


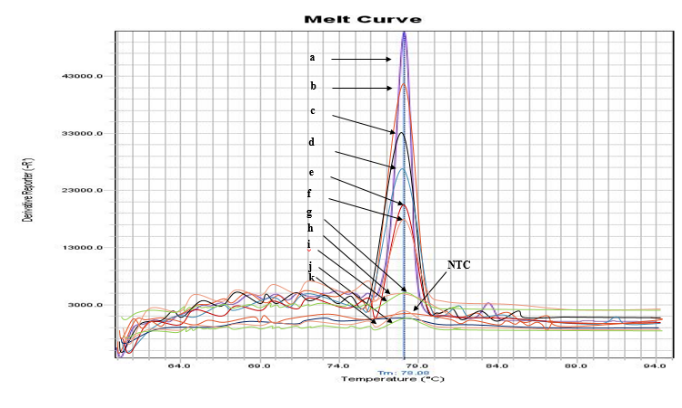

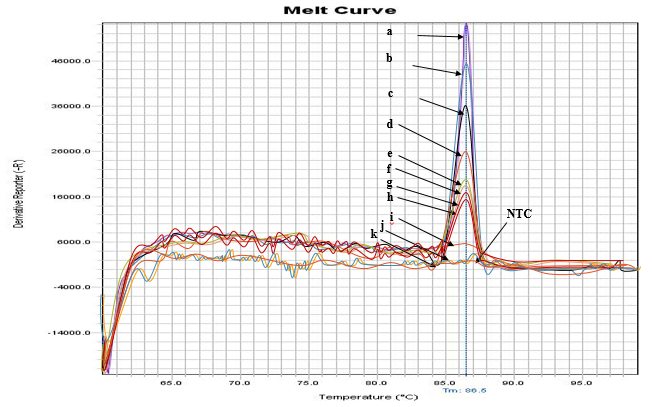

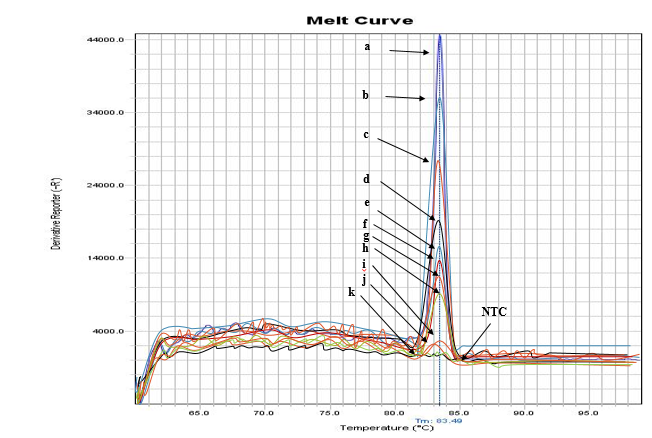

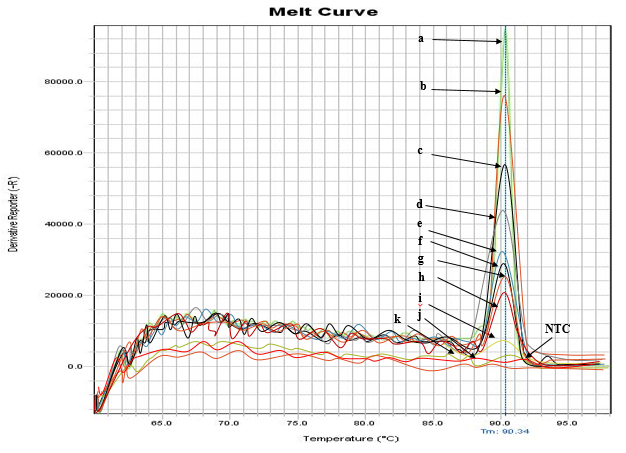


A

B

C

D


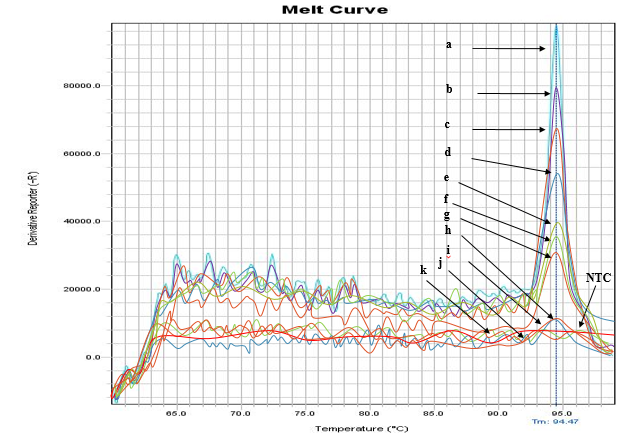


E
